# Straw Returning Strategies and Decomposing Inoculant Addition Shape Microbial Communities and Functional Potential in Black Soil

**DOI:** 10.3390/microorganisms14102220

**Published:** 2026-10-02

**Authors:** Chunting Wang, Tingting Song, Zhengyuan Fu, Tengda Yang, Wei Wang, Tianyang Zhang, Honghui Teng

**Affiliations:** Jilin Provincial Key Laboratory of Emerging Contaminants Identification and Control, Jilin Normal University, Siping 136000, China

**Keywords:** straw returning, soil nutrients, microbial community, CAZy, KEGG

## Abstract

Straw returning methods and decomposing inoculants can jointly affect soil microbial communities and their functional potential. However, the differences in microbial responses under different straw returning methods remain unclear. In this study, a 180-day incubation experiment was conducted to compare the effects of straw mulching, shallow tillage, and deep tillage, with or without decomposing inoculant addition, on soil properties, microbial communities, CAZy families, and KEGG pathways. The results showed that straw mulching maintained the highest soil moisture content, reaching 19.31%. Under shallow tillage with inoculant addition, soil total organic carbon reached 27.31 g/kg, representing an 18.58% higher than in CK. This treatment also showed relatively high microbial community richness and diversity, with enrichment of *Candidatus_Nitrosocosmicus*, *Clostridium*, and *Sphingobacterium*. Under deep tillage with inoculant addition, soil total nitrogen reached the highest value of 1.227 g/kg, and *Limnobacter*, *Candidatus_Nitrosotalea*, and *Sanguibacter* were enriched. Additionally, inoculant addition was associated primarily with changes in GT and CBM families and amino acid biosynthesis-related pathways under shallow tillage, but with changes in CE and GH families and nitrogen metabolism-related pathways under deep tillage. Soil moisture was significantly associated with community composition, while total nitrogen and available nitrogen were significantly associated with KEGG functional profiles. Overall, the effects of decomposing inoculant addition on soil nutrients, microbial communities, and functional profiles varied across straw returning methods. Soil moisture and nitrogen availability emerged as important factors associated with these microbial and functional differences.

## 1. Introduction

Northeast China hosts one of the world’s three major Mollisol regions. Its abundant black soil resources support regional agricultural production and are vital to national food security. Although black soils are rich in organic matter and nutrients, long-term intensive cultivation, residue removal, and inappropriate fertilization have accelerated organic matter depletion, weakening soil structure and nutrient supply [1,2,3]. Therefore, enhancing organic material return, improving soil nutrient conditions, and sustaining microbially mediated ecological processes are essential for black soil conservation, sustainable utilization, and green agricultural production. As the dominant residue in Northeast China’s maize systems, maize straw is rich in cellulose, hemicellulose, and nutrients such as nitrogen (N), phosphorus (P), and potassium (K), making its efficient utilization critical for resource recycling and sustainable soil management [4,5,6]. Straw returning reintroduces crop-fixed carbon and mineral nutrients into the soil, thereby promoting soil organic carbon accumulation, improving soil structure, and enhancing nutrient cycling [7,8]. However, its ecological effects depend not only on organic matter input, but also on straw distribution, straw–soil contact, and local moisture, aeration, and microbial activity [9]. Consequently, different returning methods can substantially influence straw decomposition, nutrient release, and microbial functional responses.

Previous studies have shown that straw-returning methods strongly regulate straw decomposition, nutrient cycling, and microbial responses. Compared with surface mulching, straw incorporation generally increases straw–soil contact and promotes organic matter decomposition. For example, straw incorporation promoted the decomposition of carbon functional groups, such as alkoxy C and carbonyl C, by 25–68%. It reduced cumulative soil CO_2_ emissions by 12–31%, and increased soil organic carbon, total nitrogen (TN), and microbial biomass carbon and nitrogen [4]. These results indicated strong carbon sequestration potential. Straw mulching primarily affects microbial growth and necromass accumulation by altering surface soil hydrothermal conditions and carbon–nitrogen substrate supply. In a 12-year field experiment in the Mollisol region of Northeast China, continuous maize straw mulching increased amino sugar contents in the 0–10 cm soil layer by approximately 21–45%, accompanied by greater fungal necromass accumulation and enhanced stabilization of microbial-derived carbon [10]. The amount of straw applied as mulch also influences microbial interaction networks, with 33–67% mulching producing more complex root and rhizosphere microbial community structures [11]. Moreover, no-tillage with straw mulching can increase microbial and potential metabolic diversity throughout the 0–150 cm soil profile, enhance nitrogen retention in the root zone, and reduce leaching losses [12]. Straw-returning depth is another key factor. Deep incorporation at 30 cm is more effective than shallow application at 10 cm in increasing soil organic carbon, TN, total phosphorus (TP), and total potassium (TK), likely by promoting microbial community restructuring and nutrient cycling [8]. Overall, straw-returning methods not only determine straw distribution in soil but also reshape microbial communities and ecological functions by regulating moisture, substrate contact, nutrient release, and microbial habitats.

During straw return, decomposing inoculants are widely applied to accelerate straw decomposition and nutrient release. By enhancing straw-degrading microorganisms and associated enzyme activities, functional inoculants can promote the breakdown of organic components such as cellulose and hemicellulose. For example, the low-temperature microbial consortium M44, mainly composed of *Pseudomonas*, *Brevundimonas*, and *Flavobacterium*, increased straw degradation by 8.9% and promoted the release of carbon, N, P, and K by 6.7%, 12.8%, 7.4%, and 9.6%, respectively. Its application also increased soil β-glucosidase activity and was associated with the enrichment of *Pseudomonas* and other taxa involved in straw degradation in the soil microbial community [13]. Similarly, the compound inoculant HC-2 increased cellulose degradation by 31.5% and strengthened soil transcriptional activity and ion transport-related metabolic processes [14]. Beyond directly promoting straw decomposition, inoculants can reshape soil microbial communities and functional processes. A *Bacillus*–*Trichoderma* compound inoculant increased the relative abundance of Acidobacteria to 22.7–60.8% and Sordariomycetes to 13.7–30.9% [15], while the *Streptomyces* compound inoculant G123 enriched Proteobacteria and reduced soil-borne disease risk [16]. The combined application of arbuscular mycorrhizal fungi and *Trichoderma* increased β-glucosidase activity in albic soil by 77.6% and promoted beneficial taxa such as *Sphingomonas* [17]. In addition, inoculant application can upregulate the nitrification gene AOB-*amo*A while reducing denitrification-related genes, thereby decreasing N_2_O emissions and increasing soil nitrate-N content [18]. Together, these findings suggest that decomposing inoculants regulate soil carbon and nitrogen cycling through linked processes involving accelerated straw decomposition, modified nutrient release, and microbial community restructuring.

Although the effects of straw return and decomposing inoculants on soil properties and microbial communities have been widely studied, less is known about how soil properties during incubation and microbial profiles at the experimental endpoint differ across straw placement and inoculant treatment combinations in black soil. To address this question, we conducted an incubation experiment using black soil from Northeast China. Maize straw was applied as surface mulch, incorporated at a shallow depth of 5 cm, or incorporated at a deep depth of 25 cm, with and without decomposing inoculant addition. We measured soil physicochemical properties during incubation and characterized microbial community composition and metagenomic functional potential at day 180. Our objectives were to test whether (1) soil moisture, pH, electrical conductivity, organic carbon, and nitrogen, phosphorus, and potassium levels differed among treatments during incubation; (2) microbial community composition, diversity, and metagenomic functional potential differed among treatments at day 180; and (3) soil properties were associated with microbial community composition and functional potential at the experimental endpoint. By integrating these measurements within a unified experimental framework, this study provides evidence to inform maize straw management in black soil.

## 2. Materials and Methods

### 2.1. Experimental Design

The black soil and maize straw used in this study were collected from the experimental field of Jilin Zhuoyue Shunbang Biotechnology Co., Ltd. (Jilin, China). The basic physicochemical properties of the soil are shown in Table S1. Maize straw was pretreated as described previously [19]. The microbial inoculant used in this study was Nongxuwang straw-decomposing inoculant, which contains *Bacillus subtilis*, *Bacillus amyloliquefaciens*, yeast, *Aspergillus niger*, and *Aspergillus oryzae*, as reported in reference [19]. An indoor incubation experiment was conducted in polyvinyl chloride (PVC) pots measuring 30 cm in diameter and 30 cm in height. Each pot contained 25 kg of air-dried soil. Before pot filling, soil moisture was adjusted to 16% ± 1%, approximately 60% of the soil’s maximum water-holding capacity. Maize straw was added to all treatments except CK at 0.27 kg per pot, equivalent to 10.8 g per kg of air-dried soil. For inoculant-amended treatments, the inoculant was dispersed in water and applied to the soil at 21.2 mg per pot, equivalent to 0.848 mg per kg of air-dried soil. The application rate was calculated from the dosage specified in the product instructions. Seven treatments were established with three replicate pots each: no straw addition (CK), straw mulching (FG), straw mulching with inoculant (FJ), shallow incorporation at 5 cm (QG), shallow incorporation at 5 cm with inoculant (QJ), deep incorporation at 25 cm (SG), and deep incorporation at 25 cm with inoculant (SJ). The 21 pots were randomly positioned indoors, with no blocking factor. Every five days, the pots were weighed and water was added as needed to restore the target soil moisture level.

Soil samples were collected from the same pots at 30, 60, 90, 135, and 180 days after straw return using a five-point composite sampling method. At each sampling time, a soil corer was inserted to the bottom of the soil column at five locations in each pot. Approximately 150 g of material was collected per pot. Straw fragments were retained in the collected material, and the sampled material was not returned to the pots. A portion of each fresh composite sample was used to determine soil moisture content (MC) by the gravimetric oven-drying method. Another portion was air-dried and sieved for the determination of pH, electrical conductivity (EC), total organic carbon (TOC), TN, alkali-hydrolyzable nitrogen (AN), TP, available phosphorus (AP), TK, and available potassium (AK), according to the methods described in reference [19]. The remaining portion was stored at −20 °C for subsequent molecular analysis. Soil physicochemical properties were measured at each sampling time, whereas metagenomic sequencing was performed only on samples collected at day 180.

### 2.2. Soil DNA Extraction and Metagenomic Sequencing

Soil samples collected 180 days after straw return were used for metagenomic sequencing. Total soil DNA was extracted using the FastDNA™ Spin Kit for Soil DNA (MP Biomedicals, Illkirch, France) following the manufacturer’s protocol. DNA concentration and purity were assessed with a NanoDrop 2000 spectrophotometer (Thermo Fisher Scientific, Waltham, MA, USA), and DNA integrity was evaluated by 1% agarose gel electrophoresis. DNA samples meeting library construction requirements were used for metagenomic sequencing.

Qualified DNA samples were sent to Majorbio Bio-Pharm Technology Co., Ltd. (Shenzhen, China) for library preparation and paired-end sequencing on the Illumina NovaSeq platform (Illumina, San Diego, CA, USA). Raw reads were quality-filtered to remove adapters, low-quality reads, and short sequences using Trimmomatic (v0.39; SLIDINGWINDOW:4:20 and MINLEN:50) with the following parameters: ILLUMINACLIP:adapters_path:2:30:10, SLIDINGWINDOW:4:20, and MINLEN:50. The SLIDINGWINDOW:4:20 option trimmed reads when the average quality score within a 4 bp window fell below 20, and MINLEN:50 discarded reads shorter than 50 bp after trimming. Host-derived sequences were then removed using Bowtie2 with the—very-sensitive parameter. The resulting clean reads were used for downstream taxonomic profiling and functional annotation. Sequencing output and quality metrics for each sample are provided in Table S3, including raw read counts, raw bases, GC content, read counts after cleaning, and Q20 and Q30 values before and after quality filtering.

### 2.3. Analysis of Microbial Community Composition

Microbial community composition was analyzed based on the clean reads obtained after quality control and host sequence removal. Taxonomic annotation was performed using Kraken2 (2.0.7-beta). The clean reads were aligned against a custom microbial nucleotide database, which was constructed by selecting sequences belonging to bacteria, fungi, archaea, and viruses from the NCBI NT nucleotide database and the RefSeq whole-genome database. The Kraken2 classification parameter was set to—confidence 0.2. The taxonomic classification results generated by Kraken2 were further processed using Bracken to re-estimate taxon abundance, with default parameters. Based on the Bracken output, the read counts and relative abundances of taxa were calculated at the kingdom, phylum, class, order, family, genus, and species levels. The genus-level abundance table was used for subsequent analyses of microbial community composition, diversity, and differential abundance. Non-microbialtaxa were retained in this table and were included in the subsequent community analyses.

### 2.4. CAZy and KEGG Functional Annotation

Functional annotation was performed using clean reads after quality control and host sequence removal. UniRef90 gene-family abundances were estimated using HUMAnN3 (version 3.9) and subsequently used for CAZy and KEGG annotation. Clean reads were first aligned against the UniRef90 protein database using DIAMOND (version 2.0.15), and reads failing to meet the reported filtering criteria were discarded. The main parameters were as follows: translated_query_coverage_threshold = 90.0, prescreen_threshold = 0.01, evalue_threshold = 1.0, and translated_subject_coverage_threshold = 50.0.

HUMAnN3 was used to calculate UniRef90 gene family abundances from the alignment results. Abundance tables were then normalized to copies per million (CPM) using the humann_renorm_table script with the parameter—units cpm. The normalized UniRef90 gene-family abundances were summed according to their assignments to CAZy families and KEGG Orthology groups to generate the corresponding functional abundance tables. Because a gene family may be assigned to more than one function, functional abundance tables do not necessarily sum to 1,000,000 CPM per sample.

### 2.5. Data Analysis

Line charts, bar charts, and box plots were generated using Origin 2024 (OriginLab, Northampton, MA, USA). Redundancy analysis (RDA), volcano plots, and heatmaps were generated in R software (v4.5.0) using the vegan package, ggplot2 package, and pheatmap package, respectively. For the sequencing provider’s comparisons among treatment groups, microbial abundances were assessed using Kruskal–Wallis tests followed by Dunn’s pairwise tests, with Bonferroni-adjusted *p* values reported for the pairwise comparisons. LEfSe was also used to identify group-associated taxa, with an LDA score threshold of 2. Network heatmaps were constructed using the Wecomm Bioinformatics Cloud Platform (https://www.bioincloud.tech/, accessed on 27 August 2026). Partial least squares structural equation modeling (PLS-SEM) and related statistical analyses were performed in R software (v4.5.0) to evaluate the direct and indirect relationships among variables. Key variables were selected based on variable weights and loading values to optimize the model. All variables were standardized before analysis, and normality transformations were applied when necessary. Model goodness of fit was evaluated using the goodness-of-fit (GoF) index, with GoF > 0.36 indicating good model fit.

## 3. Results

### 3.1. Dynamic Changes in Soil Physicochemical Properties Under Different Straw Returning Methods

**Figure 1:**
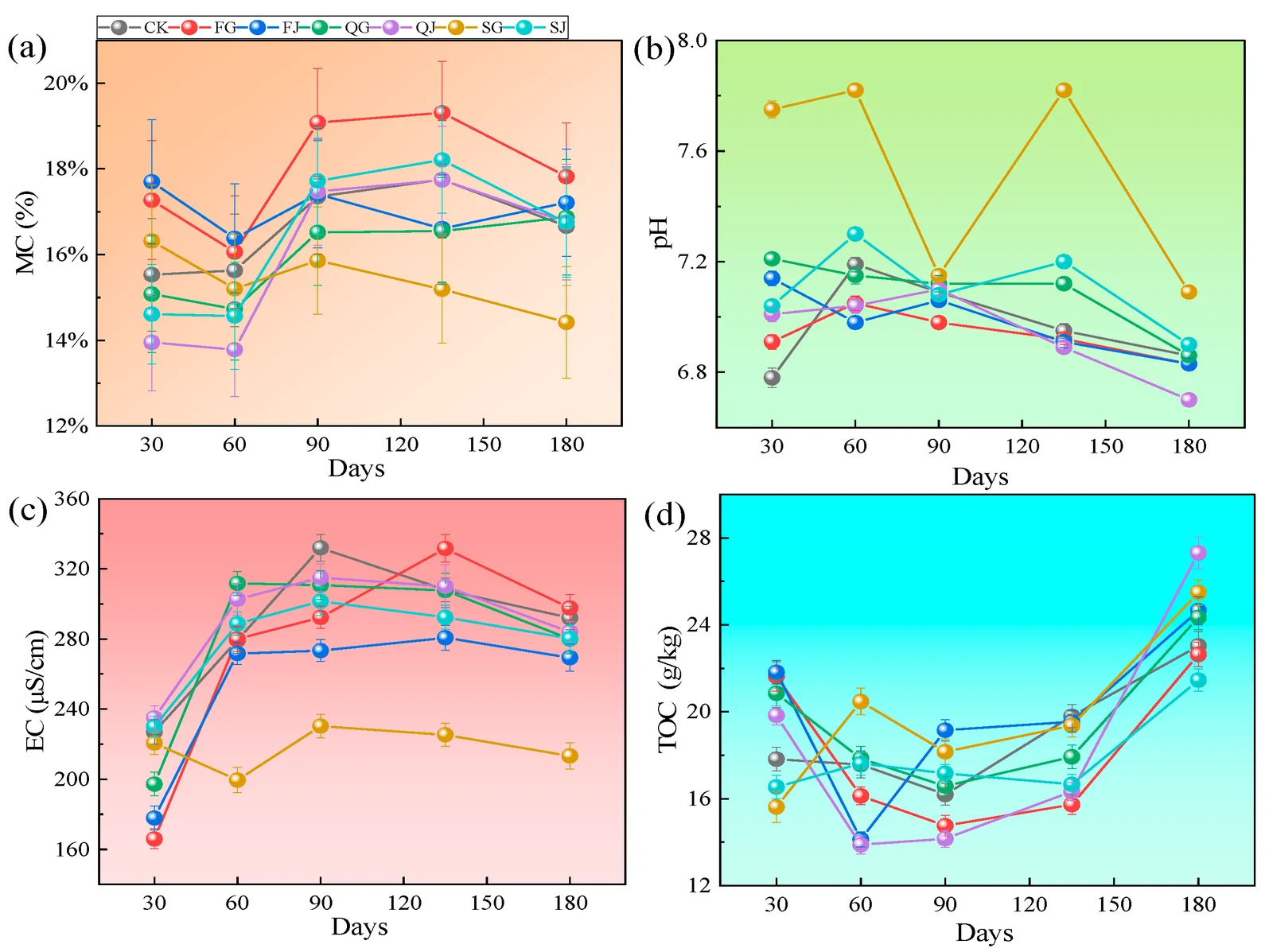
Comparative variations in soil physical and chemical properties across different treatments. (**a**) moisture content (MC), (**b**) pH, (**c**) electrical conductivity (EC), and (**d**) total organic carbon (TOC). Data are presented as means ± standard deviation (SD), with *n* = 3 biological replicates per treatment at each time point.

Different straw-returning methods induced treatment-specific and temporal changes in soil physicochemical properties (Figure 1). MC ranged from 13.78% to 19.31% across treatments (Figure 1a). At 30 days, a relatively high MC of 17.70% was observed in FJ. From 90 to 135 days, the highest MC occurred in FG, reaching 19.08% and 19.31%, respectively, indicating a strong water-retention effect of straw mulching. At 180 days, MC remained high in FG (17.82%) but decreased to 14.42% in SG. Compared with SG, higher MC was maintained at 90, 135, and 180 days in SJ, suggesting that inoculant addition partly improved soil moisture under deep incorporation. Further, pH ranged from 6.70 to 7.82 and was generally near neutral (Figure 1b). During most periods, pH remained relatively high in SG but was lower in SJ, indicating that inoculant addition may mitigate the pH increase caused by deep incorporation. By day 180, pH had decreased and values had become more similar among treatments, with the lowest value of 6.70 observed in the QJ treatment. Additionally, EC generally increased initially and then declined (Figure 1c). The highest EC were observed in QJ and SJ at 90 days, whereas the maximum EC in FG occurred at 135 days. From 60 days onward, relatively low EC was maintained in SG, indicating that deep incorporation may reduce soluble ion accumulation in surface soil.

Soil TOC fluctuated during incubation, decreasing in the early and middle stages but increasing later (Figure 1d). At 30 days, TOC reached 21.63 and 21.82 g/kg in FG and FJ treatments, respectively, but decreased in most straw-returning treatments by 60 days. At 180 days, TOC increased to 21.46–27.31 g/kg, with the highest value in QJ, which was 18.58% higher than CK. Relatively high TOC was also observed in SG, FJ, and QG. Compared with QG, TOC concentration was 12.16% higher in QJ, indicating that shallow incorporation combined with inoculant addition was associated with a higher late-stage TOC concentration, rather than demonstrating net carbon accumulation. However, lower TOC concentration was observed in SJ than in SG, suggesting that the effects of inoculant addition on TOC concentration depended on straw-returning depth and incubation stage.

**Figure 2:**
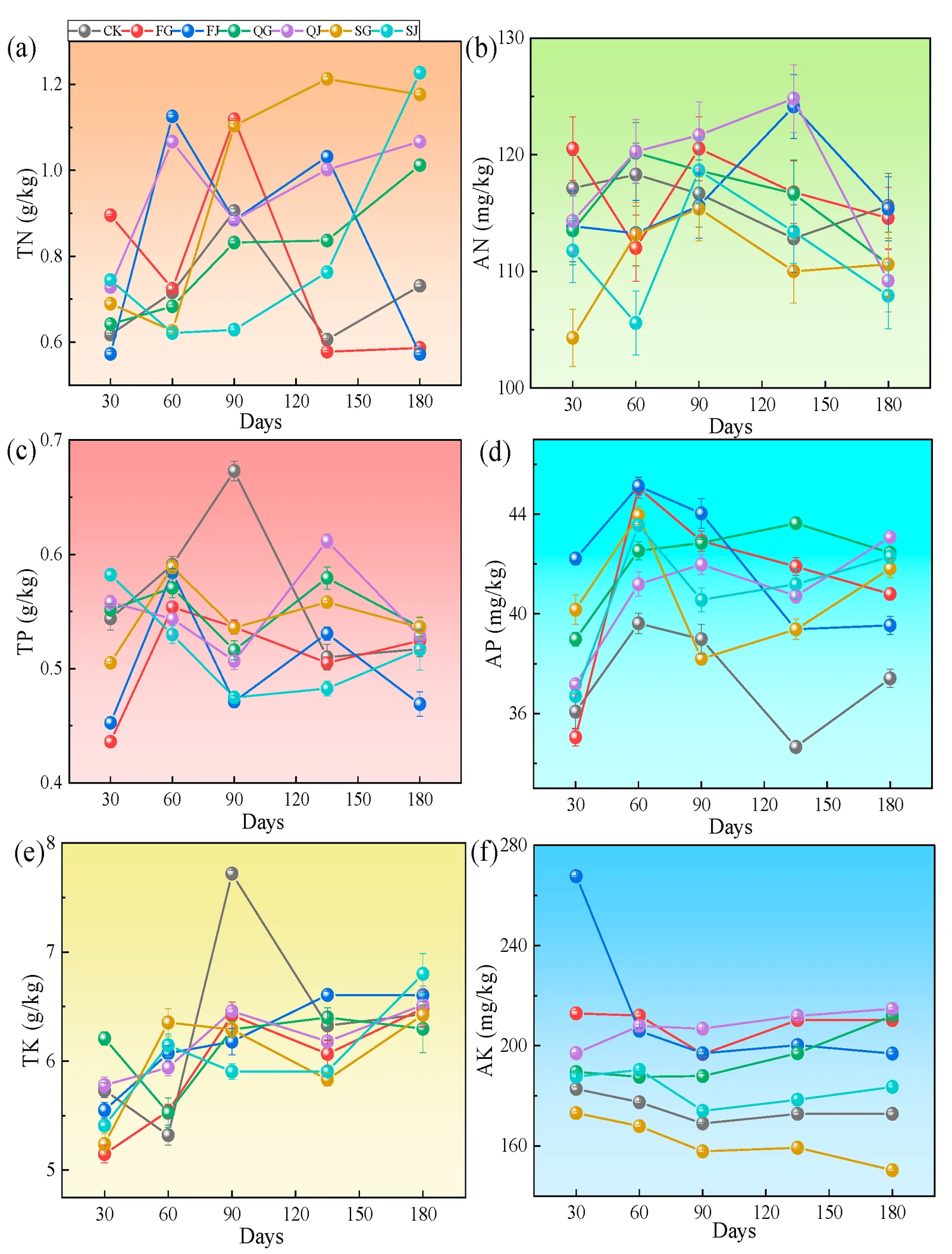
Variations in soil chemical properties under different treatments. (**a**) total nitrogen (TN), (**b**) available nitrogen (AN), (**c**) total phosphorus (TP), (**d**) available phosphorus (AP), (**e**) total potassium (TK), and (**f**) available potassium (AK). Data are presented as means ± standard deviation (SD), with *n* = 3 biological replicates per treatment at each time point.

Soil N, P, and K also showed stage-dependent responses (Figure 2). TN ranged from 0.572 to 1.227 g/kg (Figure 2a), with peak values occurring at different times among treatments. At 180 days, TN contents were 67.85%, 60.88%, 45.96%, and 38.44% higher than CK in SJ, SG, QJ, and QG, respectively, whereas lower TN contents than CK were observed in FG and FJ. These results suggested that tillage-based straw return, especially deep incorporation, was associated with higher late-stage TN concentrations, but do not establish greater nitrogen accumulation or retention. A different pattern was observed for AN (Figure 2b). Relatively high AN was maintained from 60 to 135 days in QJ, reaching a maximum of 124.83 mg/kg at 135 days. A similarly high value was also observed in FJ at this stage. However, by 180 days, AN was lower in all straw-returning treatments than in CK. This indicated that the mid-stage increase in AN was not sustained. At 180 days, the higher TN concentrations observed in some treatments did not coincide with higher AN concentrations; AN was lower than CK in all straw-returning treatments. TP fluctuated only slightly, ranging from 0.436 to 0.673 g/kg (Figure 2c), and remained between 0.469 and 0.536 g/kg at 180 days. In contrast, AP varied more markedly (Figure 2d). The highest AP was observed in FJ from 30 to 90 days, with a maximum of 45.13 mg/kg at 60 days, whereas the highest AP at 135 days occurred in QG. At 180 days, AP reached 43.08 mg/kg in QJ, which was 15.16% higher than CK and 32.76% higher than QG. These results indicated that shallow incorporation with inoculant addition helped maintain phosphorus availability during the later stage. TK ranged from 5.15 to 7.72 g/kg (Figure 2e). By 180 days, TK tended to stabilize across treatments. The highest value was observed in SJ, whereas differences among the other treatments were limited. This suggested that straw-returning method had a relatively weak effect on TK. In contrast, AK was more strongly affected by straw-returning method (Figure 2f). At 30 days, AK reached 267.66 mg/kg in FJ, which was 46.46% higher than CK. This indicated that straw mulching with inoculant addition promoted early K release. Thereafter, AK in FJ gradually declined. During the later stage, AK increased in QG and QJ, reaching 211.91 and 214.68 mg/kg at 180 days, respectively. In SG, AK decreased to 150.28 mg/kg, whereas SJ maintained a higher value of 183.53 mg/kg. This suggested that inoculant addition partly alleviated the decline in surface-soil AK under deep incorporation. Overall, straw mulching mainly enhanced water retention. Deep incorporation was associated with higher TN concentrations at 180 days, but was associated with relatively low EC and AK.

### 3.2. Effects of Different Treatments on Soil Microbial Community Diversity, Community Structure, and Differentially Abundant Genera

**Figure 3:**
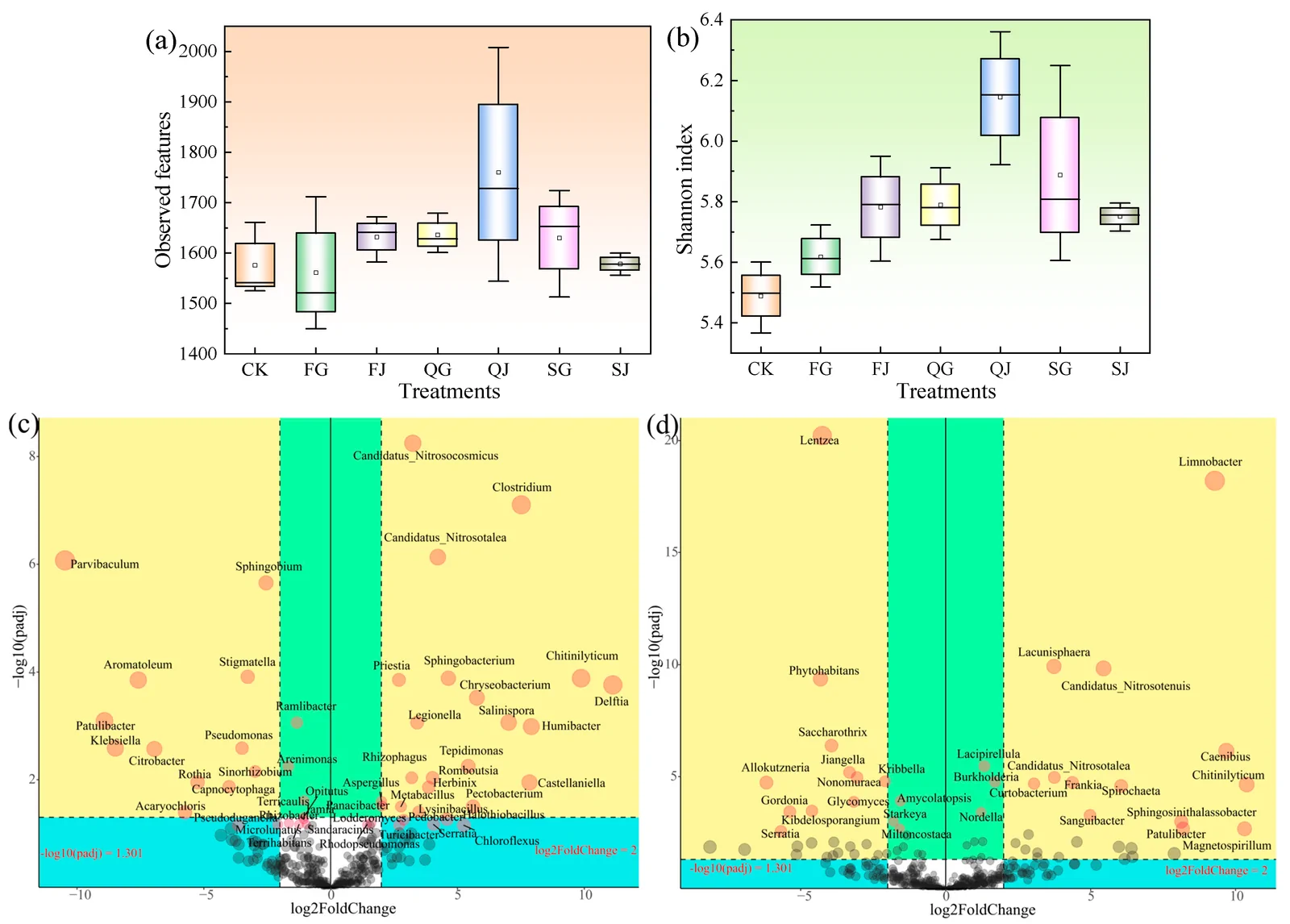
Alpha diversity and genus-level differential abundance analysis under different treatments. (**a**) Observed features, reflecting the richness of detected taxa, (**b**) Shannon index, indicating microbial community diversity by considering both richness and evenness, (**c**) volcano plot of differentially abundant genus-level taxa between the QJ and QG treatments, and (**d**) volcano plot of differentially abundant genus-level taxa between the SJ and SG treatments. Only features with an occurrence frequency of at least 0.2 were included. Differentially abundant features were determined based on an adjusted *p* value < 0.05 and |log_2_(fold change)| ≥ 2, with the top 30 significant features annotated.

Different straw-returning methods distinctly affected soil microbial alpha diversity (Figure 3). The mean observed features ranged from 1561 to 1760, following the order QJ > QG > FJ > SG > SJ > CK > FG. Compared with CK and QG, observed features increased by 11.7% and 7.6% in QJ, respectively, indicating that shallow straw incorporation combined with decomposing inoculant addition increased microbial richness. The Chao index showed the highest potential richness in SG (1753), followed by QG, QJ, and FJ, all exceeding CK (Table S2). However, observed features in QJ ranged from 1544 to 2008, indicating notable within-treatment variation. The Shannon index followed the order QJ (6.14) > SG (5.89) > QG (5.79) > FJ (5.78) > SJ (5.75) > FG (5.62) > CK (5.49). Compared with CK, the Shannon index increased by 11.9% and 7.3% in QJ and SG, respectively, indicating higher overall microbial diversity. The Simpson index and Pielou’s evenness showed similar patterns, with the highest values in QJ, followed by SG. Berger–Parker dominance was lower in QJ and SG than CK, indicating reduced dominance of abundant taxa and greater community evenness. Inoculant addition increased observed features and Shannon index under mulching and shallow incorporation but reduced these indices under deep incorporation, indicating that inoculant effects on alpha diversity depended on straw-returning method and tillage depth.

Principal coordinate analysis (PCoA) based on Bray–Curtis dissimilarity showed variation in microbial community composition among treatments (Figure S1). The first two coordinates explained 19.32% and 14.56% of the variation, respectively. PERMANOVA indicated an overall difference among treatments (R^2^ = 0.517, pseudo-F = 2.502, *p* = 0.001). In the ordination plot, QJ samples were located in the upper-right region, SG samples were positioned on the right, and CK, FG, and FJ samples occurred mainly on the left or lower side. To further characterize treatment-associated differences, genus-level differential abundance analysis was performed. Compared with CK, significant enrichment of *Candidatus_Nitrosocosmicus*, *Candidatus_Nitrosotalea*, *Legionella*, *Sphingobacterium*, *Chryseobacterium*, and *Clostridium* was observed in QJ, whereas *Parvibaculum* and *Klebsiella* were decreased (Figure S2). Compared with QG, significant enrichment of *Candidatus_Nitrosocosmicus*, *Clostridium*, *Candidatus_Nitrosotalea*, *Priestia*, *Sphingobacterium*, *Chryseobacterium*, *Legionella*, *Chitinilyticum*, *Delftia*, *Salinispora*, *Humibacter*, and *Castellaniella* was observed in QJ, whereas *Parvibaculum*, *Sphingobium*, *Aromatoleum*, *Stigmatella*, *Patulibacter*, *Klebsiella*, *Citrobacter*, and *Pseudomonas* were decreased (Figure 3c). Among these genera, *Candidatus_Nitrosocosmicus* and *Candidatus_Nitrosotalea* are archaeal taxa. The other genera listed here are bacterial taxa. *Candidatus_Nitrosocosmicus* showed the most pronounced increase, whereas *Parvibaculum* showed the clearest decrease. These results indicated that shallow incorporation with inoculant addition selectively reshaped microbial community composition.

Significant genus-level differences were also observed under deep incorporation. Compared with CK, *Lentzea*, *Glycomyces*, *Jiangella*, and *Nonomuraea* enriched significantly in SG (Figure S3), indicating that deep straw incorporation favored these genera. However, compared with SG, SJ significantly enriched *Limnobacter*, *Lacunisphaera*, *Candidatus_Nitrosotenuis*, *Candidatus_Nitrosotalea*, *Caenibius*, *Chitinilyticum*, and *Spirochaeta*, while depleting *Lentzea*, *Phytohabitans*, *Saccharothrix*, *Allokutzneria*, *Jiangella*, *Nonomuraea*, and *Kibdelosporangium* (Figure 3d). *Limnobacter* showed a marked increase, whereas *Lentzea* showed the strongest decrease. Notably, the genera enriched in SG relative to CK, including *Lentzea*, *Glycomyces*, *Jiangella*, and *Nonomuraea*, were all decreased in SJ relative to SG. Thus, inoculant addition did not simply enhance the effect of deep incorporation, but reversed the responses of some deep tillage sensitive taxa. In addition, *Limnobacter*, *Candidatus_Nitrosotalea*, and *Sanguibacter* were enriched in SJ relative to both SG and CK (Figure S4), suggesting that they may be key responsive taxa under deep incorporation with inoculant addition. Overall, decomposing inoculant addition markedly reshaped soil microbial communities under both shallow and deep straw incorporation, but the response was strongly dependent on tillage depth. In QJ, microbial responses were characterized by increased richness, diversity, and evenness, together with enrichment of *Candidatus_Nitrosocosmicus*, *Candidatus_Nitrosotalea*, *Clostridium*, and *Sphingobacterium*. In SJ, alpha diversity did not exceed SG, but clear genus-level taxonomic replacement occurred, mainly involving enrichment of *Limnobacter*, *Candidatus_Nitrosotalea*, and *Sanguibacter* and depletion of *Lentzea*, *Glycomyces*, and *Jiangella*. These findings indicated that decomposing inoculants primarily regulated microbial communities through selective effects on specific genera, and that this regulation is constrained by straw-returning depth.

### 3.3. Compositional Characteristics of Soil Microbial CAZy Under Different Treatments

**Figure 4:**
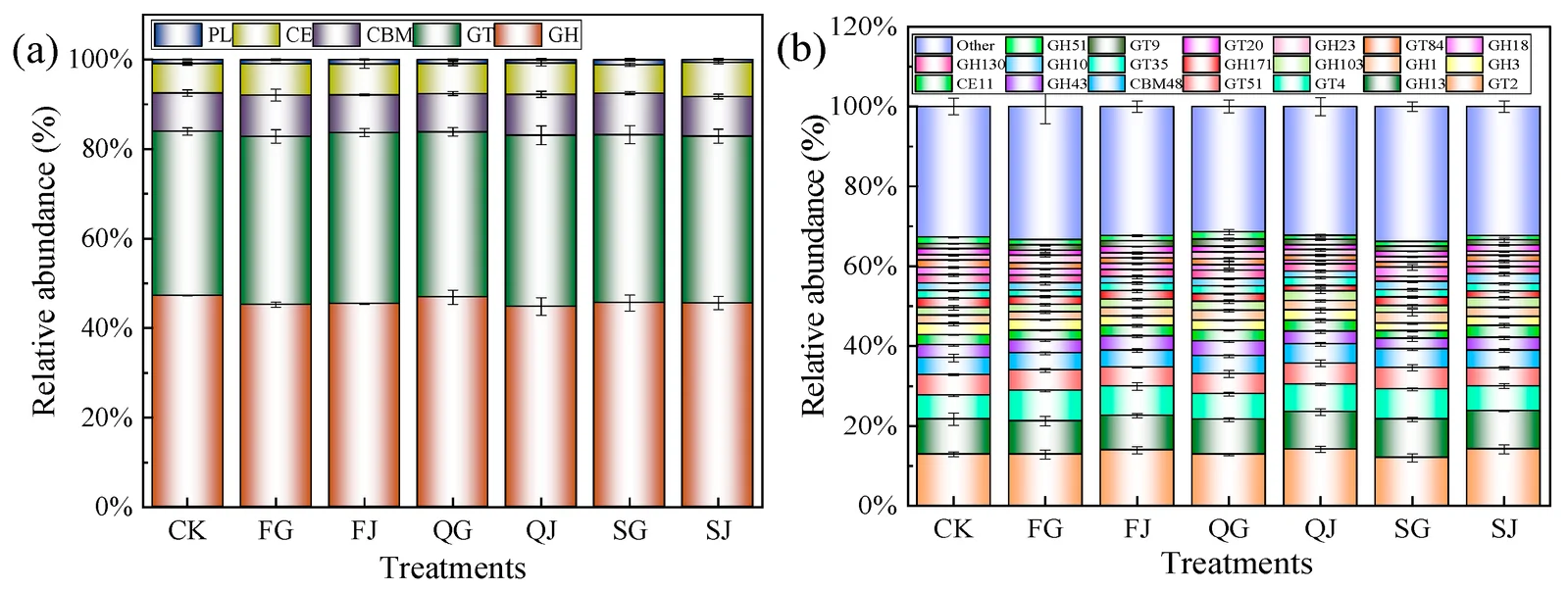
CAZy-based functional composition and abundance profiles under different treatments. (**a**) Relative abundance distribution at the CAZy class level, (**b**) relative abundance profiles of CAZy families.

CAZy annotation showed that soil microbial CAZy-annotated gene profiles were generally similar among treatments, although the relative abundances of specific classes and families varied (Figure 4). GH and GT dominated all treatments, with a combined relative abundance of 82.82–83.95%, making them the two largest classes of CAZy annotations. GH accounted for 44.85–47.30% and was generally lower in straw-returning treatments than in CK, with the lowest value under QJ. The relative abundance of GT accounted for 36.65–38.25%, with the highest value observed in FJ. Compared with QG, GH showed a relative decrease of 2.07% in QJ, whereas GT, CBM, and CE showed relative increases of 1.29%, 0.60%, and 0.30%, respectively. Similar GH and GT abundances were observed between SG and SJ, whereas CE showed a relative increase of 1.32% in SJ, accompanied by lower relative abundances of CBM and PL. Here and below, between-treatment percentage changes refer to changes relative to the stated reference treatment, not percentage-point differences. These results describe broadly similar class-level profiles, with differences in the observed relative abundances of individual classes between the shallow and deep incorporation treatments.

At the family level, a few dominant CAZy families accounted for high proportions, whereas the remaining families were more dispersed (Figure 4b). GT2 was the most abundant family across treatments, accounting for 12.06–14.20%. Compared with the corresponding non-inoculated treatments, GT2 showed relative increases of 1.05%, 1.23%, and 2.14% in FJ, QJ, and SJ, respectively, with the largest reported relative increase in SJ. GH13 was the second dominant family, accounting for 8.34–9.63%, with relatively high proportions in SG and SJ. GT4 and GT51 accounted for 5.98–7.62% and 4.49–5.36%, respectively, showing variation among GT families despite limited variation at the class level. CBM48 was the dominant CBM family, accounting for 4.18–4.81%, with the highest value in QJ. Compared with QG, CBM48 showed a relative increase of 0.34% in QJ, consistent with the observed class-level increase in CBM. GH43 ranged from 2.57% to 3.70%. CE11 reached 3.00% in SJ, representing a relative increase of 1.04% compared with SG. This pattern was observed alongside the higher relative abundance of the CE class in SJ.

Further analysis of GH families showed that several GH families had lower relative abundances in QJ than in QG. In particular, the relative abundances of GH51, GH171, and GH18 were 1.82%, 1.94%, and 1.37% in QG, compared with 0.98%, 1.35%, and 0.91% in QJ, respectively, consistent with the overall lower relative abundance of GH in QJ. Thus, the observed difference in GH class abundance was accompanied by differences in the relative abundances of multiple GH families. Under deep incorporation, total GH abundance was similar between SG and SJ, but the relative abundances of individual families differed. The relative abundances of GH43, GH103, GH10, and GH130 were higher in SJ, whereas those of GH1, GH171, and GH18 were lower. Notably, GH18 accounted for 2.29% in SG and 1.36% in SJ, illustrating that similar class-level relative abundances can coexist with differences at the family level. The “Other” category accounted for 31.42–33.84%, representing CAZy families grouped rather than displayed individually. Overall, GH and GT consistently dominated the CAZy-annotated gene profiles, whereas the observed relative abundances of some families varied among treatments. In QJ, the observed differences included higher relative abundances of GT2, GT4, GT51, and CBM48 and lower relative abundances of several GH families. In SJ, the observed differences included higher relative abundances of GT2 and CE11, together with differences in the relative abundances of GH families. These descriptive results indicate differences in the relative distribution of CAZy-associated genes. They do not establish statistically significant treatment effects on individual CAZy classes or families, nor do they directly demonstrate changes in enzyme activity or cellulose or hemicellulose decomposition.

### 3.4. Functional Characteristics of Soil Microbial Communities Based on KEGG Annotation Under Different Treatments

At KEGG level 1, the relative abundance profiles of annotated microbial functions were broadly similar among treatments (Figure S5). Metabolism was the dominant category, accounting for 73.93–74.60% of total relative abundance, followed by genetic information processing (9.72–10.60%). Cellular processes, human diseases, and organismal systems accounted for 5.15–5.51%, 4.74–5.06%, and 2.74–2.91%, respectively. The lowest relative abundance of metabolism was observed in SJ, whereas genetic information processing was highest in SG and lowest in FJ. Overall, the level 1 profiles showed limited variation among treatments; differences in the plotted relative abundances were more apparent at finer levels of KEGG classification.

**Figure 5:**
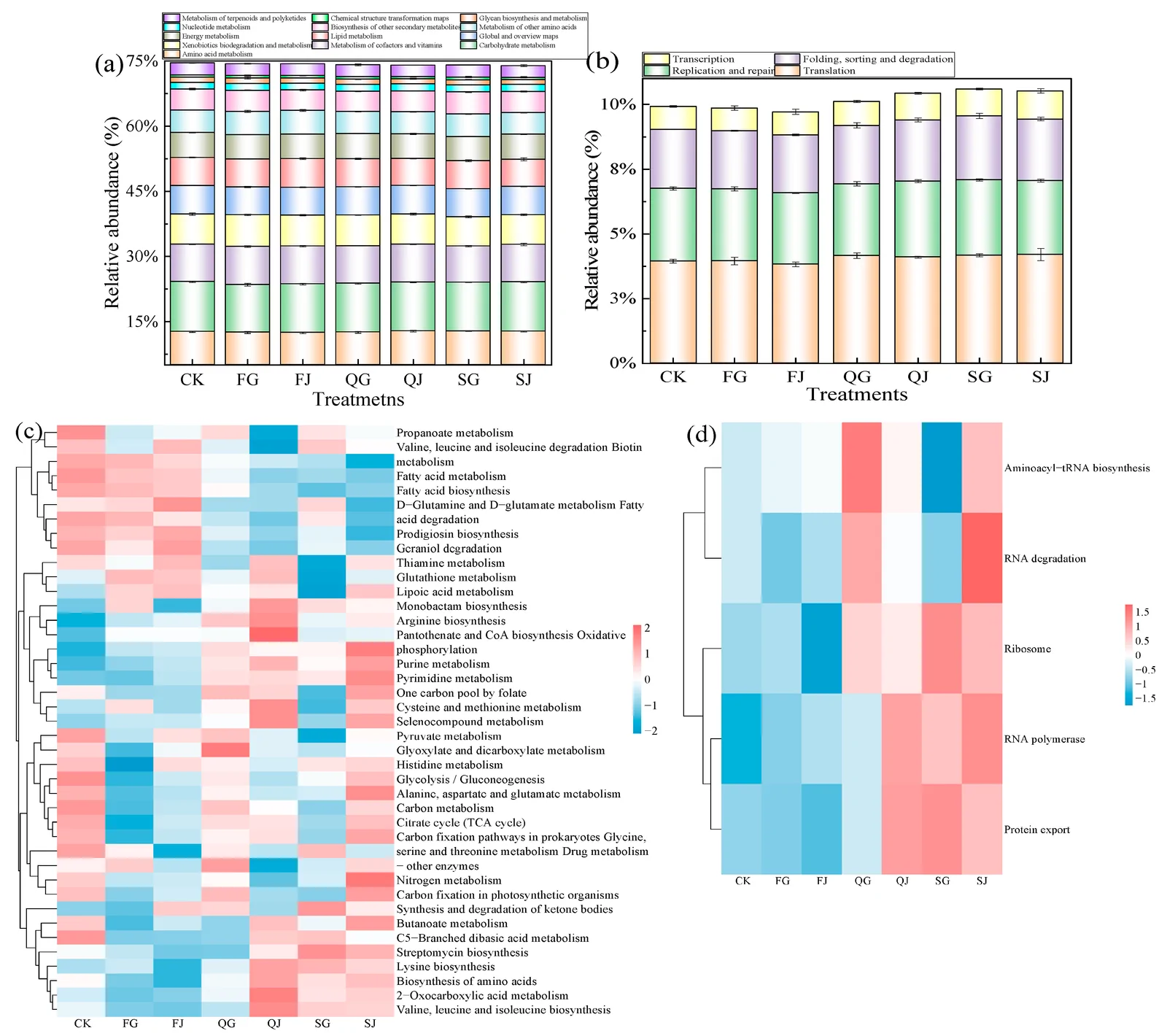
KEGG-based functional profiles under different treatments. (**a**) level 2 pathways within metabolism, (**b**) level 2 pathways within genetic information processing, (**c**) level 3 pathways within metabolism, and (**d**) level 3 pathways within genetic information processing.

At KEGG level 2, the relative abundances of some metabolic categories varied among treatments (Figure 5a). Amino acid metabolism and carbohydrate metabolism were the most abundant categories, accounting for 12.46–12.82% and 10.97–11.50%, respectively. Amino acid metabolism accounted for 12.82% in QJ and had a higher relative abundance than in QG. Relatively high values were also observed in SG and SJ. Carbohydrate metabolism had the highest relative abundance in CK and was generally lower in the straw-returning treatments. Its relative abundance was 10.97% and 11.19% in the treatments without and with inoculant addition under straw mulching, respectively, and 11.26% and 11.37% under deep incorporation; similar values were observed between QG and QJ. The relative abundance of metabolism of cofactors and vitamins ranged from 8.36% to 8.78% and was higher in the inoculated treatments than in their corresponding non-inoculated treatments. Xenobiotics biodegradation and metabolism showed the opposite descriptive pattern. Other categories, including lipid metabolism, energy metabolism, and metabolism of other amino acids, varied only slightly. Within genetic information processing, translation was the most abundant category, accounting for 3.83–4.20% (Figure 5b). Its relative abundance was higher in QG, QJ, SG, and SJ than in CK, FG, and FJ, with the highest value observed in SJ. Replication and repair had a higher relative abundance in QJ than in QG, while transcription had higher relative abundances in QJ than in QG and in SJ than in SG. These observations describe differences in annotated functional proportions, not measured rates of translation, transcription, or replication.

At KEGG level 3, differences in the relative abundances of individual annotated pathways were observed (Figure 5c). In QJ, valine, leucine and isoleucine biosynthesis, biosynthesis of amino acids, lipoic acid metabolism, pantothenate and CoA biosynthesis, 2-oxocarboxylic acid metabolism, arginine biosynthesis, and lysine biosynthesis accounted for 3.18%, 1.45%, 1.96%, 1.32%, 1.31%, 0.92%, and 0.73%, respectively; all had higher relative abundances than in QG (Figure S6). Propanoate metabolism, fatty acid metabolism, fatty acid degradation, and histidine metabolism had lower relative abundances in QJ than in QG. Thus, the annotated pathway profiles differed between QJ and QG, particularly among pathways classified under amino acid and cofactor metabolism; these data do not establish changes in metabolic activity. Under deep incorporation, oxidative phosphorylation, purine metabolism, pyrimidine metabolism, nitrogen metabolism, and carbon fixation in photosynthetic organisms accounted for 0.85%, 0.69%, 0.91%, 0.71%, and 1.28% in SG, compared with 0.87%, 0.71%, 0.94%, 0.72%, and 1.37% in SJ, respectively. The difference in nitrogen metabolism was particularly small (0.71% versus 0.72%) and should not be interpreted as evidence of increased nitrogen metabolic activity. C5-branched dibasic acid metabolism and fatty acid degradation had lower relative abundances in SJ than in SG, whereas butanoate metabolism was slightly higher. These comparisons characterize the relative distribution of KEGG annotations under deep incorporation without establishing biologically meaningful changes in pathway activity.

KEGG level 3 pathways within genetic information processing included aminoacyl-tRNA biosynthesis, RNA degradation, ribosome, RNA polymerase, and protein export (Figure 5d). RNA polymerase and protein export accounted for 0.86% and 0.76% in QG, compared with 0.92% and 0.81% in QJ, respectively (Figure S6). RNA degradation and RNA polymerase accounted for 0.85% and 0.90% in SG, compared with 0.89% and 0.93% in SJ, respectively. Overall, some level 3 pathways showed differences in relative abundance among treatments, although these comparisons alone do not establish statistically significant or biologically consequential changes. The KEGG results describe functional potential inferred from annotation; they do not directly demonstrate changes in gene expression or metabolic activity.

### 3.5. Coupling Relationships and Potential Pathways Among Soil Environmental Factors, Microbial Communities, and Functional Potential

**Figure 6:**
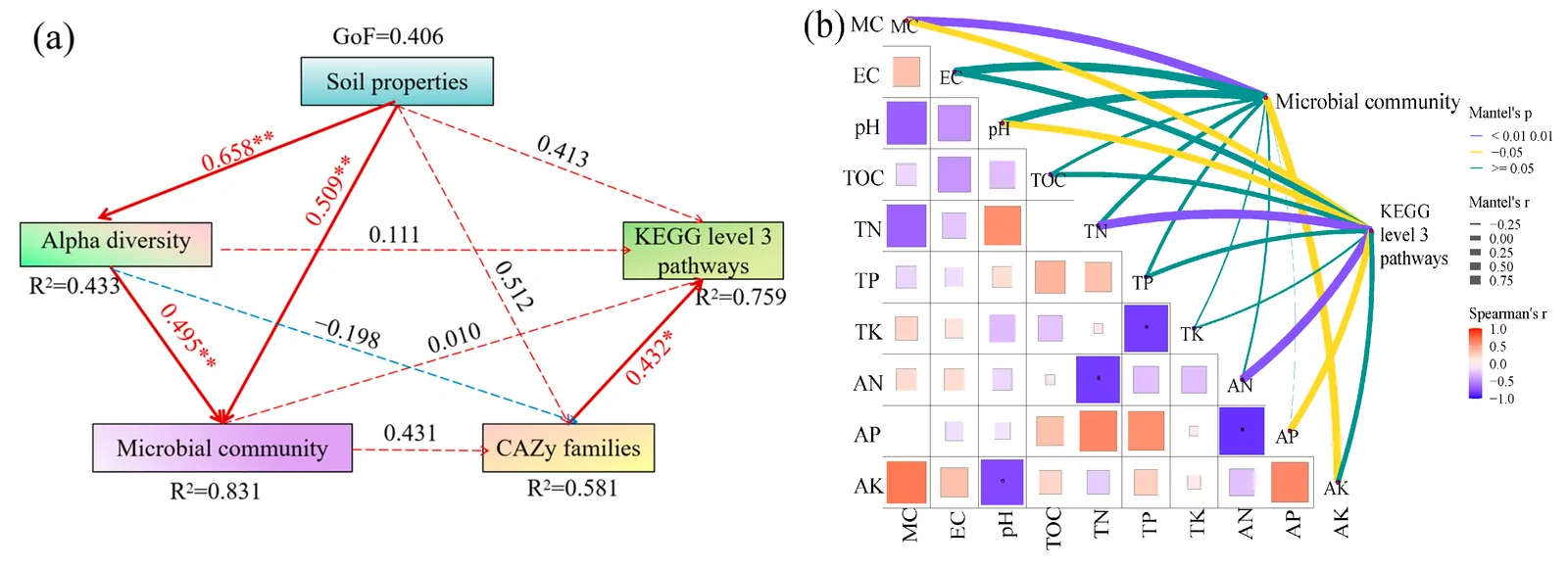
Integrated modeling and network analysis of microbial community and functional profile variations under different treatments. (**a**) PLS-PM integrating soil properties with alpha diversity, microbial community composition, CAZy families, and KEGG level 3 pathways to resolve their multivariate linkages. The PLS-PM analysis included all 21 soil samples from seven treatments (three biological replicates per treatment); no samples were excluded. Standardized path coefficients are displayed beside the arrows, and R^2^ values denote the variance explained for each endogenous variable. Model performance was assessed using GoF index. (**b**) Network heatmap showing the associations of microbial communities and KEGG level 3 pathways with soil properties. Spearman’s correlation coefficients characterize pairwise associations, while Mantel tests evaluate matrix-level relationships between microbial or functional profiles and soil properties. Line colors indicate Mantel test significance levels: purple, *p* < 0.01, yellow, *p* < 0.05; and green, *p* ≥ 0.05. Significance levels are indicated as * *p* < 0.05, ** *p* < 0.01.

PLS-SEM was used to examine associations among soil properties, microbial alpha diversity, microbial community composition, CAZy family profiles, and KEGG level 3 pathway profiles (Figure 6a). Soil properties were positively associated with alpha diversity and microbial community composition, and alpha diversity was positively associated with community composition. The model explained 43.3% of the variance in alpha diversity and 83.1% of the variance in microbial community composition. The latter reflects the predictors included in the specified model, including soil properties and alpha diversity, rather than the contribution of soil properties alone.

The direct paths from soil properties to CAZy families and KEGG level 3 pathways were not significant. Correlation analysis also identified associations between individual CAZy families and soil physicochemical properties (Figure S7). GT2 was positively correlated with TK, whereas GH13 was negatively correlated with MC and positively correlated with TN. CBM48 was negatively correlated with AN and positively correlated with AP, while GH171 showed the opposite directions of association with AN and AP. GH3 was positively correlated with EC, and GT9 was positively correlated with AK. These correlations describe covariation between soil properties and annotated CAZy family profiles.

In the PLS-SEM model, the path coefficient from microbial community composition to CAZy families was 0.431, whereas the direct path coefficient from community composition to KEGG level 3 pathways was 0.010. CAZy families were positively associated with KEGG level 3 pathways (path coefficient = 0.432, *p* < 0.05). The paths from alpha diversity to CAZy families and KEGG level 3 pathways were not significant. Together, these results describe associations among the variables within the specified model; they do not establish a sequential causal pathway from soil properties through microbial communities and CAZy families to KEGG pathways.

Mantel tests identified associations between soil properties and microbial community composition or KEGG level 3 pathway profiles (Figure 6b). MC was associated with microbial community composition. KEGG level 3 pathway profiles were associated with several soil properties, including TN, AN, MC, pH, and AP, whereas associations involving EC, TOC, and TP were comparatively weak. These patterns suggested that soil properties and microbial profiles varied together across the samples. Because both may also vary with experimental treatment, the observed associations may partly reflect shared treatment-related differences rather than independent mechanistic relationships. Overall, the PLS-SEM and Mantel results provide complementary descriptions of covariation among soil conditions, microbial community characteristics, and annotated functional profiles, without demonstrating direct causal effects.

## 4. Discussion

### 4.1. Effects of Different Straw Returning Methods on Soil Moisture, Carbon Accumulation, and Nutrient Dynamics

In this study, straw returning showed clear temporal variation in soil physicochemical properties, with patterns differing among returning methods. During the middle and late incubation stages, MC was higher under straw mulching than under deep incorporation. At 180 days, it was 17.82% in FG and 14.42% in SG (Figure 1a). This difference was consistent with greater surface-soil moisture under mulching, although the underlying water-balance processes were not measured. Straw mulching can reduce cumulative soil evaporation by 3.08–9.9%, with stronger inhibition as decomposition progresses due to increased mulch-layer resistance, while decomposition-derived organic matter can enhance soil water-holding capacity, especially within the medium to high suction range [20]. Similarly, strip mulching was reported to increase soil water storage in the 0–60 cm layer by 15.0–19.3%, whereas deep incorporation caused smaller and more variable changes, ranging from −5.0% to 3.8% [21]. Thus, the absence of a continuous surface mulch layer under deep incorporation might partly explain the lower MC in SG than FG. Higher MC in SJ than SG at 90, 135, and 180 days was also observed, but its cause could not be determined from MC measurements alone. Changes in straw decomposition, microbial communities, and soil conditions remain possible explanations [22,23]. Changes in pH and EC further indicated stage-dependent chemical patterns following straw returning. The relatively high pH in SG at earlier sampling times was followed by a decline across treatments, while EC generally increased initially and then decreased (Figure 1b,c). This pattern might reflect early base cation release during straw decomposition, followed by acidification associated with organic matter mineralization and nitrogen transformation, while initial soil pH also influenced the direction of pH change [24,25]. These processes provide possible explanations for the pH pattern but were not directly distinguished in this study. The timing of peak EC differed among treatments, which described changes in soluble-ion concentrations rather than directly measured nutrient-release rates. N, P, and K in straw can be continuously released within 90 days, with maximum release rates of 48.3%, 60.3%, and 72.1%, respectively, and burial depth affects these processes [25]. The relatively low EC in SG from 60 days onward, together with low surface-soil AK, was consistent with lower soluble-ion concentrations in the sampled layer, but did not establish a lower rate of nutrient release.

Soil TOC fluctuated during the early and middle stages but generally increased later. At 180 days, TOC in QJ was 18.58% and 12.16% higher than in CK and QG, respectively (Figure 1d). Straw returning generally increased cropland SOC, but the magnitude depended on initial soil properties, crop type, returning method, and duration [26]. Long-term experiments also showed that straw input increased total SOC and altered particulate organic carbon, dissolved organic carbon, and microbial biomass carbon [27]. The higher TOC concentration in QJ might reflect differences in straw–soil contact, the distribution of straw-derived material within the sampled layer, or organic matter composition. Inoculant-associated changes in decomposition are another possibility, but decomposition was not directly quantified; a higher TOC concentration alone did not establish an increase in soil carbon stock. TN and AN did not change synchronously. At 180 days, TN was higher in several incorporation treatments than in CK, whereas AN was lower than in CK across all straw-returning treatments (Figure 2a,b). The contrast concerned measured nitrogen concentrations, not demonstrated nitrogen retention. Straw returning improved nitrogen retention mainly by increasing organic nitrogen fractions such as amino acid nitrogen [28], but this did not necessarily increase AN. In the present study, microbial immobilization was one possible explanation for the contrasting TN and AN patterns. Increased carbon availability might stimulate microbial AN immobilization into biomass or organic nitrogen fractions, leading to divergent TN and AN trends [6]. Differences in the distribution of straw-derived nitrogen or in the proportions of organic and inorganic nitrogen were also possible. Because these processes were not directly measured, the higher TN concentrations could not be attributed specifically to enhanced decomposition or nitrogen retention.

TP showed smaller treatment differences than AP. At 180 days, AP reached 43.08 mg/kg in QJ, 32.76% higher than in QG (Figure 2c,d). This was a difference in measured AP concentration, not direct evidence of a particular phosphorus-transformation process. Different forms of straw returning could alter soil phosphorus fractions and phosphatase activity, but AP responses varied with straw form and soil conditions [29,30]. Therefore, the higher AP in QJ might be related to differences in straw placement or phosphorus transformations, although these were not directly assessed. AK showed an early peak in FJ and remained higher in QG and QJ than in CK at 180 days. A previous study showed that maximum release rates of N, P, and K from straw reached 48.3%, 60.3%, and 72.1%, respectively, after 90 days of inoculant treatment, and that straw burial depth affected straw decomposition and nutrient release [25]. The observed AK patterns were consistent with differences in potassium availability among treatments, although the contribution of potassium released from straw could not be determined from soil AK concentrations alone. Overall, straw-returning methods and inoculant addition were associated with distinct temporal patterns in surface-soil moisture and measured carbon and nutrient concentrations. Differences in straw placement and decomposition may have contributed to these patterns, but their relative contributions remained uncertain.

### 4.2. Differential Effects of Straw Returning Depth and Decomposing Inoculant Addition on Microbial Diversity and Community Composition

Straw returning was associated with changes in the soil conditions experienced by microbial communities, including organic substrate availability, MC, pH, and nutrient status. In this study, the observed features and Shannon index in QJ were 11.7% and 11.9% higher than those in CK, respectively. The Simpson index and Pielou’s evenness were also highest in QJ, whereas the Berger–Parker dominance was lower than that in CK (Figure 3a,b and Table S2). These results indicated that microbial richness, diversity, and evenness tended to be higher in QJ. Previous analyses showed that straw returning generally increased soil bacterial richness, but the response of diversity was regulated by returning duration, straw input amount, nitrogen fertilization level, and soil conditions [31]. Different straw returning methods might also have different effects on bacterial and fungal diversity [14,32]. In the present study, the response to inoculant addition likewise differed among straw-returning methods, with observed features and Shannon index values being higher in FJ than in FG and in QJ than in QG, but lower in SJ than in SG. Previous studies have shown that decomposing inoculants could affect indigenous microorganisms by promoting straw decomposition and altering soil microbial community composition, but their effects were regulated by soil texture, nutrient status, and application method [23,33]. A meta-analysis integrating 335 studies also showed that microbial inoculation generally did not significantly alter soil microbial diversity, although it clearly changed community structure and bacterial composition [34]. Together, these studies provide a context for the absence of a uniform diversity response to inoculant addition, despite the observed differences in community composition.

Genus-level differences provided a more specific view of the community response, but did not by themselves establish the functions performed by the responsive taxa. Compared with QG, significant enrichment of the archaeal genera, *Candidatus_Nitrosocosmicus* and *Candidatus_Nitrosotalea*, alongside bacterial genera including *Clostridium*, *Sphingobacterium*, *Chryseobacterium*, and *Chitinilyticum*, was observed in QJ, whereas other genera decreased (Figure 3c). These results indicated that inoculant addition did not uniformly increase all taxa, but instead altered their relative abundances under shallow straw incorporation. During straw decomposition, microbial communities can change with decomposition time and substrate composition [35,36]. Different crop straws might also form distinct lignocellulose-degrading microbial communities and CAZy functional combinations [37]. Therefore, the differentially abundant genera in QJ might be associated with changes in the straw-related habitat and substrate conditions, although substrate use by these taxa was not determined in this study. The enrichment of *Candidatus_Nitrosocosmicus* and *Candidatus_Nitrosotalea* warranted separate consideration as an archaeal, rather than exclusively bacterial, community response. Previous studies showed that some species within these two genera had ammonia-oxidizing potential, and their distribution was usually closely related to soil pH, ammonium nitrogen content, and oxygen conditions [38,39]. Considered alongside the AN pattern during the middle stage of incubation, the higher relative abundances of these archaeal genera in QJ might reflect changes in the soil nitrogen environment. However, their enrichment alone did not demonstrate increased ammonia oxidation or nitrification activity. In addition, significant increases in *Clostridium*, *Sphingobacterium*, and *Chitinilyticum* were detected in QJ. Some species within these genera have been reported to utilize cellulose, hemicellulose, or other complex organic substrates [40,41]. During straw decomposition, substrate composition and availability change over time, potentially favoring taxa with different resource-use traits. The enrichment of these genera in QJ might therefore reflect differences in substrate conditions under shallow incorporation with inoculant addition, although their substrate use was not directly measured.

Significant enrichment of *Lentzea*, *Glycomyces*, *Jiangella*, and *Nonomuraea* was observed in SG relative to CK. After decomposing inoculant addition, these taxa were significantly decreased in SJ, whereas *Limnobacter*, *Candidatus_Nitrosotalea*, *Sanguibacter*, and *Chitinilyticum* were significantly enriched (Figure 3d). These opposite abundance changes indicated that decomposing inoculant addition did not simply strengthen the community characteristics observed under deep straw incorporation, but instead changed the relative abundances of different archaeal and bacterial taxa. A previous study showed that exogenous microbial inoculation could increase straw decomposition rates and reshape the community composition, interaction networks, and related functional genes of indigenous lignocellulose-degrading microorganisms, with different microbial groups showing distinct responses [23]. Meanwhile, straw input could affect soil microbial community assembly by changing carbon and nitrogen substrate conditions, leading to the replacement of taxa with different ecological strategies and accompanying shifts in metabolic functional composition [42]. Therefore, the increase in *Limnobacter* and related genera and the decrease in *Lentzea* and related genera in SJ might reflect inoculant-induced changes in resource-use conditions and community selection in deep incorporation. In addition, alpha diversity indices were lower in SJ than SG, but clear genus-level differentiation was observed between these two treatments. In addition, alpha diversity indices were lower in SJ than in SG, but clear genus-level differentiation was observed between these two treatments. Thus, diversity indices and genus-level composition captured different aspects of the response under deep incorporation. Overall, the effects of decomposing inoculants on soil microbial communities differed by straw-returning depth, with shallow and deep incorporation showing distinct patterns of diversity and taxonomic relative abundance, without establishing specific functions for the responsive genera.

### 4.3. Associations Among Microbial Communities, CAZy and KEGG Functions, and Soil Environmental Factors

The high-level CAZy and KEGG annotation profiles remained generally stable across treatments, whereas differences were observed in the relative abundances of selected CAZy families and KEGG pathways. CAZy annotation showed that GH and GT were the dominant classes, with a combined relative abundance of 82.82–83.95% (Figure 4a). The CAZy database classifies carbohydrate-active enzymes and related modules associated with the synthesis, degradation, and recognition of carbohydrates on the basis of sequence similarity [43]. The predominance of GH and GT therefore described the distribution of annotated carbohydrate-related genes, rather than measured hydrolytic or transferase activities.

At KEGG level 1, metabolism and genetic information processing accounted for the largest proportions, with only minor differences among treatments (Figure S5). This broad similarity did not preclude differences among more specific annotations. Previous studies indicated that microbial community structure changed during straw decomposition with decomposition stage and straw chemical composition [44], and that straw returning could alter soil bacterial diversity, community composition, and functional profiles [31,45]. In the present study, variation among selected CAZy families and KEGG level 3 pathways was more apparent than variation among the broad categories, although the biological importance of individual differences could not be established from relative abundances alone. Soil environmental factors were associated with microbial community and functional profiles. In the specified SEM, soil properties were associated with alpha diversity and community composition (Figure 6a), while the Mantel test identified an association between MC and microbial community composition (Figure 6b). Soil microbial communities were commonly regulated by pH, organic carbon, moisture, and nutrient availability [46], and soil structure could further affect microbial functions by regulating the transport of water, oxygen, and nutrients [47]. In this study, mulching, shallow incorporation, and deep incorporation differed in straw placement and soil conditions. MC might therefore have contributed to the observed community differences, but its association with community composition could also reflect their shared responses to treatment.

Changes in microbial community composition were associated with differences in CAZy family profiles. In the SEM, the path coefficient from microbial community composition to CAZy families was 0.431. This coefficient described a relationship within the specified model; it did not establish that changes in community composition caused changes in CAZy families. Compared with QG, QJ showed differences in the relative proportions of GH, GT, CBM, and CE. Compared with SG, SJ showed differences in CE11 and several GH families (Figure 4b). GH, GT, CE, and CBM include genes annotated to families associated with glycosidic bond hydrolysis, glycosyl transfer, carbohydrate ester modification, and substrate binding, respectively [43,48]. These patterns were consistent with treatment-associated variation in carbohydrate-related functional potential, but they did not demonstrate differences in enzyme activity or straw degradation.

The correlations between CAZy families and soil physicochemical indicators provided further evidence of covariation, rather than independent effects of individual soil properties. GT2 was positively correlated with TK, and GT9 was positively correlated with AK, whereas GH13 was negatively correlated with MC and positively correlated with TN. CBM48 was negatively correlated with AN and positively correlated with AP, while GH171 showed the opposite pattern with AN and AP (Figure S7). Thus, broad GH or GT proportions alone did not capture all of the variation among annotated families. Nevertheless, these correlations could partly reflect treatment-related differences shared by soil properties and functional profiles. This interpretive caution is relevant when comparing the present patterns with previous findings that straw decomposition involved multiple microbial groups and enzyme systems whose relative contributions varied with decomposition stage and environmental conditions [44,49].

KEGG annotation profiles also varied alongside soil properties. The Mantel test indicated associations of KEGG level 3 profiles with TN, AN, MC, pH, and AP (Figure 6b), indicating that N and available nutrients were important factors shaping metabolic pathway composition. The relative abundances of valine, leucine and isoleucine biosynthesis, biosynthesis of amino acids, lipoic acid metabolism, pantothenate and CoA biosynthesis, arginine biosynthesis, and lysine biosynthesis were higher in QJ than QG. RNA polymerase and protein export also increased (Figure 5c). These results suggested that shallow incorporation combined with inoculant addition was associated with higher relative abundances of annotations related to amino acid biosynthesis, cofactor metabolism, and genetic information processing. Oxidative phosphorylation, purine metabolism, pyrimidine metabolism, nitrogen metabolism, and carbon fixation in photosynthetic organisms had higher annotated relative abundances in SJ than in SG, while RNA degradation and RNA polymerase showed slight increases (Figure 5d). These differences suggested variation in the KEGG functional profile under deep incorporation with inoculant addition. However, the difference in the nitrogen metabolism category was small and did not establish a change in nitrogen metabolic activity. Straw returning could affect microbial functional profiles through changes in carbon and nitrogen inputs, nutrient status, and community composition [31,49]. In this study, the relatively high TN in SJ at 180 days, together with the increased relative abundance of nitrogen metabolism, suggested consistency between soil nitrogen accumulation and nitrogen metabolism-related functional configuration.

Furthermore, PLS-SEM revealed a significant positive path from CAZy families to KEGG level 3 pathways, suggesting coordinated variation between carbohydrate-active enzyme families and broader metabolic pathways. In contrast, the direct path coefficient from microbial community composition to KEGG level 3 pathways was only 0.010, whereas the path from community composition to CAZy families was stronger. Within the model, community composition was more closely associated with CAZy family profiles than with KEGG level 3 pathways. The paths from alpha diversity to CAZy families and KEGG pathways were not significant, further indicating that functional differences could not be explained simply by richness or evenness, but were more likely determined by specific microbial taxa and their functional gene combinations. Overall, straw-returning methods and decomposing inoculant addition did not substantially alter the high-level CAZy and KEGG functional framework. Instead, treatment-related differences were more apparent in specific CAZy families and KEGG pathways, alongside variation in soil properties and microbial community composition. Among environmental factors, MC was most closely associated with community composition, whereas TN and AN were strongly linked to KEGG level 3 profiles. These findings suggested that treatment-related differences in functional profiles were more apparent at finer levels of annotation than at the level of broad functional categories.

## 5. Conclusions

This study showed that soil moisture and nutrient concentrations varied with straw-returning method and incubation stage. Straw mulching had the highest observed MC, reaching 19.31%. After 180 days, shallow incorporation with decomposing inoculant addition showed 18.58%, 15.16%, and 24.25% higher TOC, AP, and AK concentrations, respectively, whereas deep incorporation with inoculant addition showed a 67.85% higher TN concentration. Shallow incorporation with inoculant addition was associated with higher microbial richness and diversity and enrichment of *Candidatus_Nitrosocosmicus*, *Clostridium*, and *Sphingobacterium*. Deep incorporation with inoculant addition enriched *Limnobacter*, *Candidatus_Nitrosotalea*, and *Sanguibacter*, while reducing *Lentzea* and *Glycomyces*. CAZy and KEGG annotations showed differences in GH, GT, and CBM proportions and amino acid biosynthesis-related pathways under shallow incorporation, and in CE, GH families, and nitrogen metabolism-related pathways under deep incorporation. The specified model explained 83.1% of the variation in microbial community composition. MC was associated with community composition, while TN and AN were associated with KEGG profiles. Overall, shallow and deep incorporation with inoculant addition showed distinct soil and microbial responses during the 180-day indoor incubation.

## Data Availability

Data will be made available on request.

## Supplementary Materials

The following supporting information can be downloaded at: https://www.mdpi.com/article/10.3390/microorganisms14102220/s1, Figure S1. Principal coordinate analysis (PCoA) of bacterial community composition based on Bray–Curtis dissimilarity. Each point represents one sample; colors indicate treatments (CK, FG, FJ, QG, QJ, SJ, and SG). PCoA1 and PCoA2 explain 19.32% and 14.56% of the variation, respectively. Overall differences among treatments were assessed by PERMANOVA (R^2^ = 0.517, pseudo-F = 2.502, *p* = 0.001); Figure S2: Volcano plot of differential microbial features between QJ and CK treatments. Features with a minimum occurrence frequency ≥0.2 were included in the analysis. Differential features were identified using a corrected *p* value threshold of 0.05 and a log_2_(fold change) threshold of 2. The top 30 significant features are labeled; Figure S3: Volcano plot of differential microbial features between SG and CK treatments. Figure S4: Volcano plot of differential microbial features between SJ and CK treatments; Figure S5: Relative abundance distribution of KEGG level 1 pathways in different treatments; Figure S6: Relative abundance of KEGG pathways at Level 3 across all treatments; Figure S7: Spearman correlation heatmap between the relative abundances of CAZy families and soil properties. Only correlations with absolute correlation coefficients (|R|) ≥ 0.6 are shown. The color scale represents the correlation coefficient, with red indicating positive correlations and blue indicating negative correlations. Asterisks denote statistically significant correlations (* *p* < 0.05, ** *p* < 0.01); Table S1: Basic physical and chemical properties of black soil; Table S2: Alpha diversity indices of microbial communities in all treatments. Table S3: Quality metrics of sequencing data output in different treatments.
