# Supplementary material for: Straw Returning Strategies and Decomposing Inoculant Addition Shape Microbial Communities and Functional Potential in Black Soil
Source: Microorganisms. 2026 Oct 2;14(10):2220. doi: 10.3390/microorganisms14102220 (PMC13707655; doi:10.3390/microorganisms14102220)
Supplement: Supplementary file 1 [file microorganisms-14-02220-s001.zip › microorganisms-4590308-supplementary.pdf]

Supplementary material for

Straw returning strategies and decomposing inoculant addition shape microbial communities and functional potential in black soil

Chunting Wang, Tingting Song*, Zhengyuan Fu, Tengda Yang, Wei Wang, Tianyang Zhang, Honghui Teng*

[1] Jilin Provincial Key Laboratory of Emerging Contaminants Identification and Control, Jilin Normal University, Siping, Jilin 136000, China; 13944040013@163.com (C.W.); stt2209017@jlnu.edu.cn (T.S.); fuzhengyuan99@163.com (Z.F.); 19971350217@163.com (T.Y.); wangwei1@jlnu.edu.cn (W.W.); zhangtiany1997@foxmail.com (T.Z.); tenghonghui@jlnu.edu.cn (H.T.)

* Correspondence: stt2209017@jlnu.edu.cn (T.T. Song); tenghonghui@jlnu.edu.cn (H.H. Teng)

Total number of pages: 11

Number of figures: 7

Number of tables: 3

Table S1 Basic physical and chemical properties of black soil

Properties	Black soil
pH	6.81±0.02
MC (%)	16.12%±0.70
EC (μS/cm)	203±5.16
TOC (g/kg)	17.11±0.70
TN (g/kg)	0.57±0.03
TP (g/kg)	0.59±0.01
TK (g/kg)	5.81±0.05
AN (mg/kg)	118±2.85
AP (mg/kg)	38.09±0.37
AK (mg/kg)	180±0.79

Table S2 Alpha diversity indices of bacterial communities in all treatments

Treatments	Chao index	Simpson index	Berger-Parker dominance index	Pielou evenness
CK	1717±107	0.862±0.007	0.357±0.009	0.517±0.009
FG	1646±154	0.878±0.010	0.329±0.024	0.530±0.007
FJ	1726±52	0.871±0.019	0.348±0.028	0.542±0.015
QG	1741±50	0.879±0.016	0.334±0.027	0.542±0.011
QJ	1739±137	0.908±0.005	0.289±0.007	0.570±0.010
SG	1753±140	0.902±0.007	0.286±0.015	0.552±0.026
SJ	1677±9	0.886±0.003	0.324±0.005	0.541±0.003

Table S3. Quality metrics of sequencing data output in different treatments

Sample ID	Raw Reads	Raw Base (GB)	%GC	Raw Q20 (%)	Raw Q30 (%)	Clean Reads	Cleaned (%)	Clean Q20 (%)	Clean Q30 (%)
CK1	25146368	7.54	64	97.96	94.34	24066598	95.71	99.07	96.55
CK2	26293135	7.89	64	97.97	94.35	25108214	95.49	99.08	96.56
CK3	25504801	7.65	64	97.83	93.91	24143608	94.66	98.98	96.22
FG1	22114129	6.63	65	97.98	94.26	21049642	95.19	99.04	96.41
FG2	26987058	8.1	64	98.08	94.53	25819754	95.67	99.09	96.58
FG3	22861325	6.86	64	98.02	94.4	21842045	95.54	99.07	96.51
FJ1	25651811	7.7	65	97.97	94.25	24426411	95.22	99.04	96.41
FJ2	30139664	9.04	64	97.99	94.32	28659897	95.09	99.06	96.49
FJ3	32573140	9.77	65	98.03	94.36	31105189	95.49	99.05	96.45
QG1	23659623	7.1	64	98.04	94.42	22595019	95.5	99.07	96.51
QG2	25414044	7.62	64	98.07	94.51	24294953	95.6	99.08	96.55
QG3	24428449	7.33	64	98.01	94.38	23311579	95.43	99.07	96.53
QJ1	28504668	8.55	61	98.19	94.62	27284039	95.72	99.07	96.45
QJ2	25601599	7.68	62	97.86	93.87	24073728	94.03	98.97	96.15
QJ3	21869345	6.56	63	98.11	94.52	20878291	95.47	99.07	96.5
SG1	24462960	7.34	64	97.98	94.2	23198660	94.83	99.03	96.36
SG2	21114570	6.33	63	98.06	94.2	20051903	94.97	98.99	96.15
SG3	21614272	6.48	64	98.03	94.32	20594340	95.28	99.04	96.38
SJ1	26242720	7.87	63	98.06	94.37	25016729	95.33	99.04	96.38
SJ2	23439975	7.03	62	98.36	95.01	21248713	90.65	99.19	96.83
SJ3	22815635	6.84	63	98.09	94.38	21746589	95.31	99.03	96.35

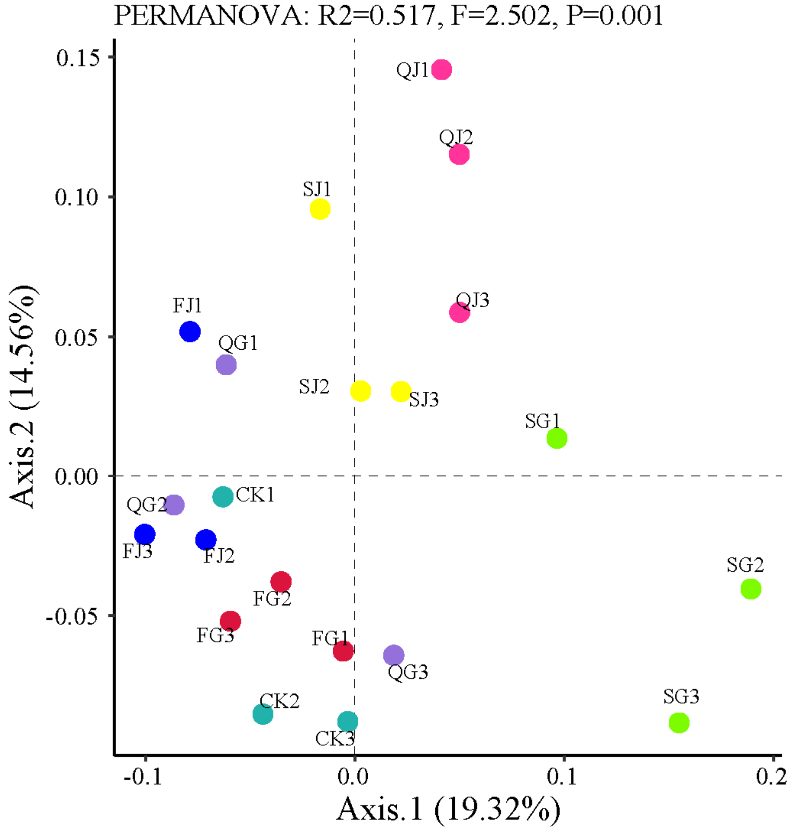

Figure S1. Principal coordinate analysis (PCoA) of bacterial community composition based on Bray–Curtis dissimilarity. Each point represents one sample; colors indicate treatments (CK, FG, FJ, QG, QJ, SJ, and SG). PCoA1 and PCoA2 explain 19.32% and 14.56% of the variation, respectively. Overall differences among treatments were assessed by PERMANOVA (R^2 = 0.517, pseudo-F = 2.502, P = 0.001).

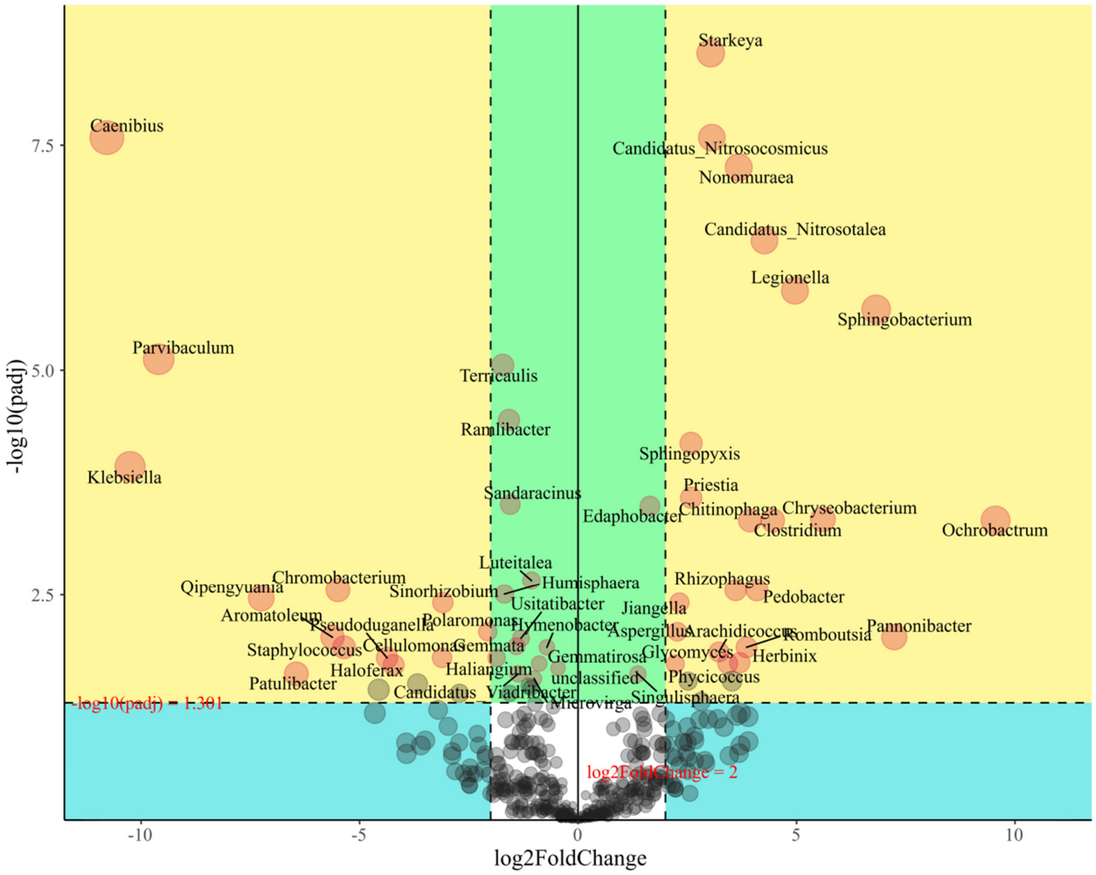

Figure S2. Volcano plot of differential microbial features between QJ and CK treatments. Features with a minimum occurrence frequency ≥ 0.2 were included in the analysis. Differential features were identified using a corrected *P* value threshold of 0.05 and a $\log_2$(fold change) threshold of 2. The top 30 significant features are labeled.

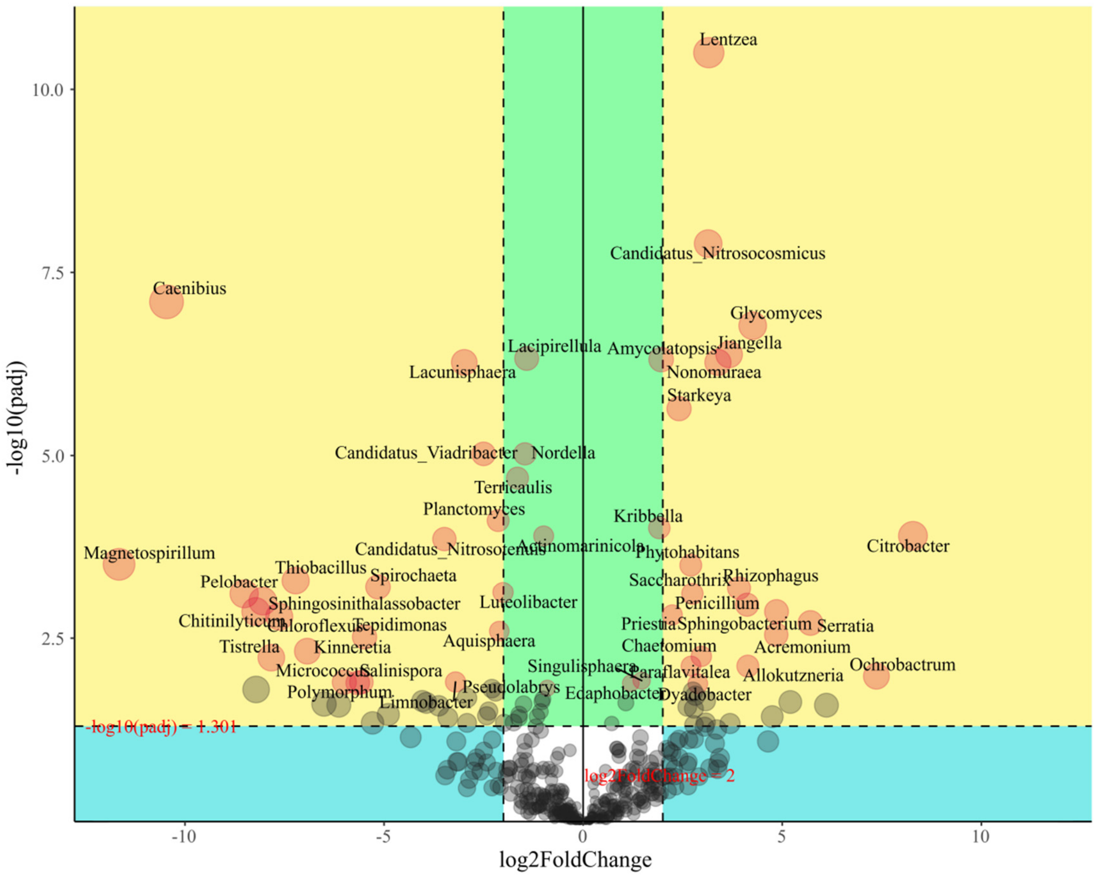

Figure S3. Volcano plot of differential microbial features between SG and CK treatments.

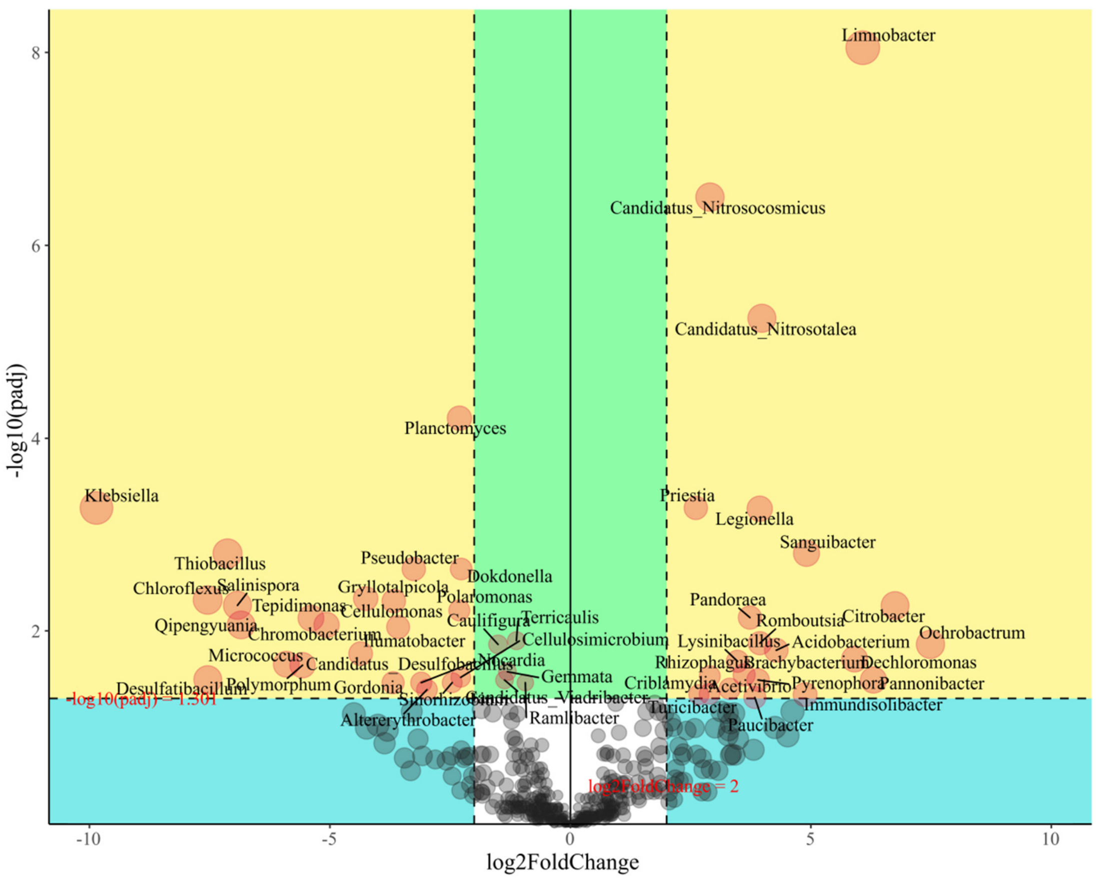

Figure S4. Volcano plot of differential microbial features between SJ and CK treatments.

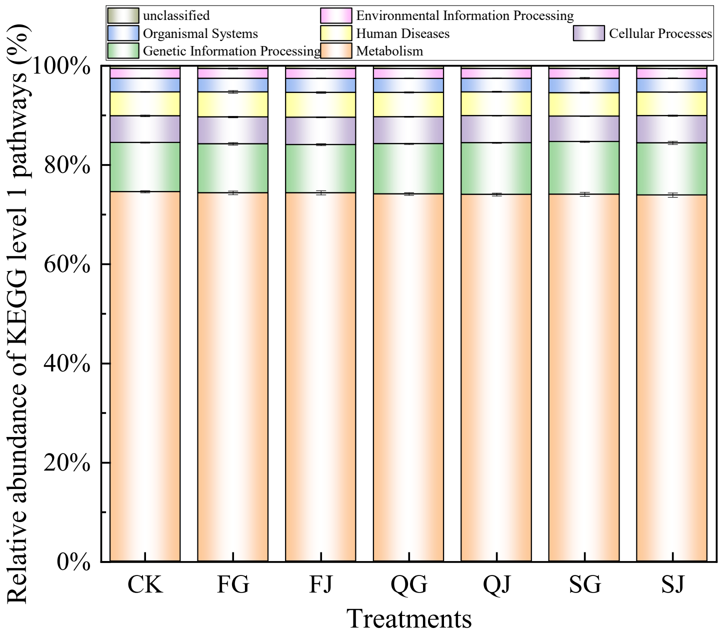

Figure S5. Relative abundance distribution of KEGG level 1 pathways in different treatments.

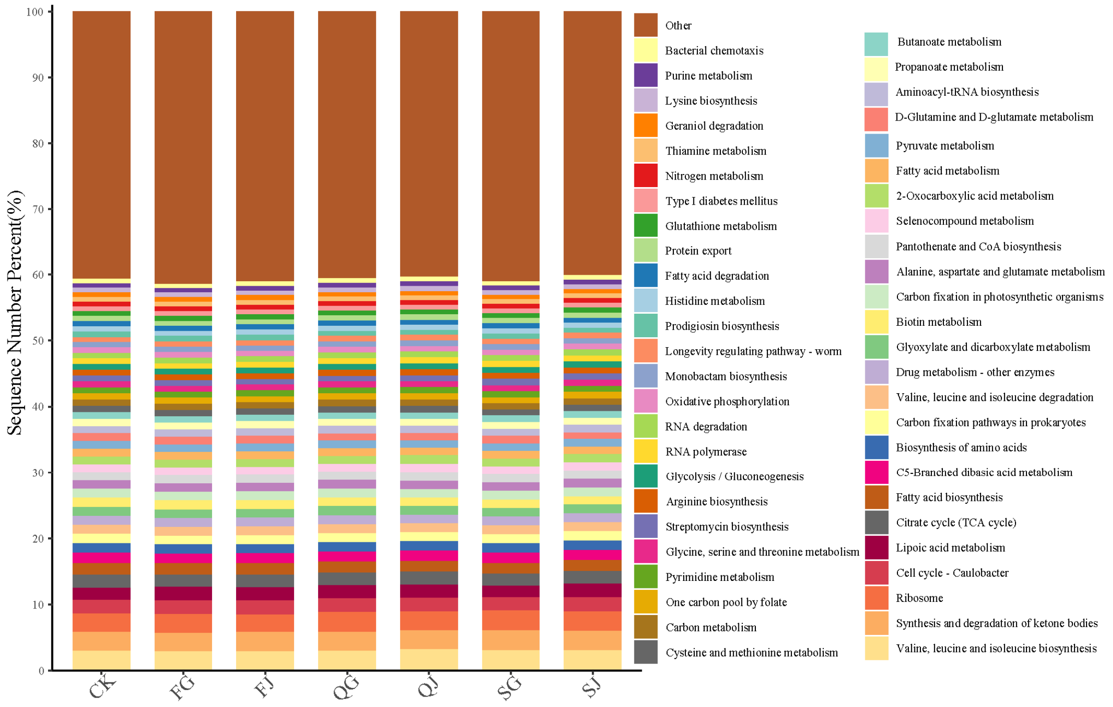

Figure S6. Relative abundance of KEGG pathways at Level 3 across all treatments

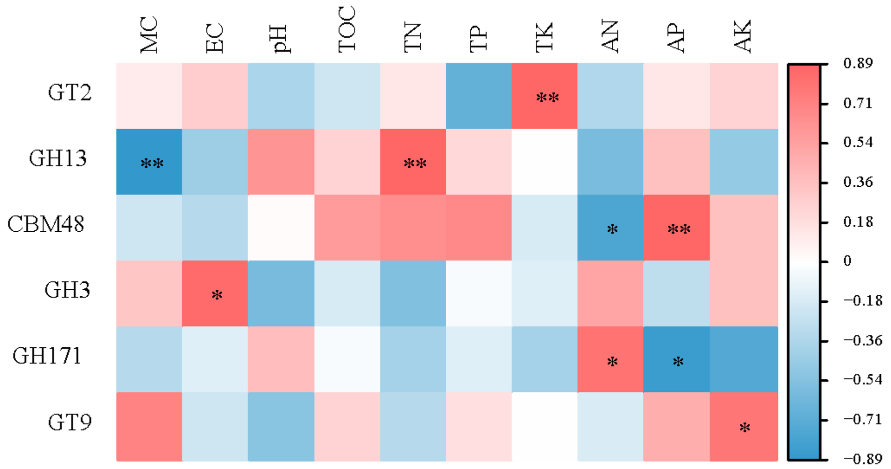

Figure S7. Spearman correlation heatmap between the relative abundances of CAZy families and soil properties. Only correlations with absolute correlation coefficients (|R|) ≥ 0.6 are shown. The color scale represents the correlation coefficient, with red indicating positive correlations and blue indicating negative correlations. Asterisks denote statistically significant correlations (*$p < 0.05$, **$p < 0.01$).
